# Biomarkers of standard criteria and marginal donor lungs during ex vivo lung perfusion: A comparative study

**DOI:** 10.1016/j.jhlto.2026.100555

**Published:** 2026-04-04

**Authors:** M.A. Hu, Z.L. Zhang, R.F. Hoffmann, C.T. Gan, E.A.M. Verschuuren, C. Van De Wauwer, H.G.D. Leuvenink, M.E. Erasmus

**Affiliations:** a Department of Cardiothoracic Surgery, University Medical Center Groningen, Groningen, Netherlands; b Department of Pulmonary Diseases and Lung Transplantation, University Medical Center Groningen, Groningen, Netherlands; c Department of Surgery, University Medical Center Groningen, Groningen, Netherlands

**Keywords:** Ex vivo lung perfusion, Biomarkers, Coagulation, Fibrinolysis, Inflammation, Glycocalyx, Endothelium

## Abstract

**Background:**

Ex vivo lung perfusion (EVLP) is a well-established method for assessing and reconditioning donor lungs before lung transplantation (LTx). Remarkably, clinical outcomes between standard criteria and marginal donor lungs after EVLP are similar despite differences in lung quality. Therefore, we explored several biomarkers during EVLP in this study. Novel findings could guide future interventions to enhance lung quality and expand the donor pool.

**Methods:**

EVLP was performed for ≥180 min with 18 standard criteria (logistical) and 15 marginal donor lungs. The biomarkers were measured in the perfusate after 90 and 180 min: d-dimer, Prothrombin Fragment 1+2 (F1+2), Plasminogen Activator Inhibitor-1 (PAI-1), urokinase Plasminogen Activator Receptor (uPAR), IL-1β, IL-6, IL-8, TNF-α, syndecan-1, hyaluronan and Vascular Cell Adhesion Molecule-1 (VCAM-1).

**Results:**

In the logistical and marginal group, 16/18 and 12/15 were transplanted, respectively. All biomarkers increased significantly during EVLP. Remarkably, d-dimer, F1+2, uPAR, IL-6, IL-8, syndecan-1, hyaluronan, and VCAM-1 were significantly higher in the marginal group. Declined donor lungs had significantly higher levels of syndecan-1 and hyaluronan than all transplanted donor lungs. Lung function during EVLP and primary graft dysfunction (PGD) were similar between the transplanted standard and marginal lungs. No significant correlations with PGD were observed.

**Conclusion:**

Marginal donor lungs sustained more injury as reflected in higher levels of fibrin degradation, inflammation, glycocalyx shedding, and endothelial activation. In addition, declined donor lungs exhibited significantly higher glycocalyx shedding. Therefore, both logistical and marginal donor lungs could potentially benefit from thrombolysis, inflammation-reducing therapy, and endothelial preservation during EVLP to enhance lung quality.

## Background

Lung transplantation (LTx) remains the sole treatment for patients with end-stage lung failure. Unfortunately, the demand for donor lungs consistently exceeds the available supply. Ex vivo lung perfusion (EVLP) has emerged as a well-established technique in recent years to assess and recondition marginal donor lungs and is currently widely adopted by many lung transplant centers to expand the donor lung pool.^1^, ^2^, ^3^ Beyond the role of assessing and reconditioning marginal donor lungs, EVLP can also safely extend the total preservation time, allowing for elective and daytime LTx procedures.^4^

Criteria for marginal donor lungs are: 1) Persistent donor partial pressure of oxygen (pO_2_) < 40 kPa with a fraction of inspired oxygen (FiO_2_) of 1.0 and a positive end-expiratory pressure (PEEP) of 5 cmH_2_O, 2) presence of lung edema, hemorrhage, contusion, or pulmonary emboli 3) Evidence of microbiological, radiographic, bronchoscopic, or intraoperative macroscopic abnormalities, and/or 4) doubtful lung function.^2^, ^5^, ^6^, ^7^ Other donor characteristics, such as high age or multiple blood transfusions, may also be considered.^8^ Conventional LTx of marginal donor lungs has been associated with increased primary graft dysfunction (PGD) and lower survival rates.^9^ However, it has been reported that marginal donor lungs assessed with EVLP result in similar clinical outcomes compared to LTx with standard criteria donor lungs.^3^, ^10^, ^11^, ^12^ Interestingly, a few studies have observed more adverse events immediately postoperatively in cases of marginal donor lungs following EVLP.^13^, ^14^

Our center utilizes EVLP routinely for both the assessment and reconditioning of marginal donor lungs and for logistical purposes with standard criteria donor lungs. Previously, we reported that standard-criteria donor lungs outperformed marginal donor lungs during EVLP.^4^ In this follow-up study, we explored the biomarker profile during EVLP of not only marginal donor lungs but also standard criteria donor lungs. We measured the following established biomarkers in a single study as an indication for fibrinolysis, coagulation, inflammation, glycocalyx injury, and endothelial activation: d-dimer, Prothrombin Fragment 1+2 (F1+2), Plasminogen Activator Inhibitor-1 (PAI-1), urokinase Plasminogen Activator Receptor (uPAR), Interleukin-1β (IL-1β), interleukin-6 (IL-6), interleukin-8 (IL-8), and Tumor Necrosis Factor-α (TNF-α), syndecan-1, hyaluronan, and Vascular Cell Adhesion Molecule-1 (VCAM-1). We hypothesize that marginal donor lungs have sustained more injury than standard criteria donor lungs, as their lung function is initially subpar. By measuring multiple biomarkers representing different injury pathways, these analyses may provide valuable insights into the management of standard-criteria and marginal donor lungs during EVLP, potentially improving graft quality and clinical outcomes.

## Materials and methods

### Study groups

EVLP procedures were performed with en bloc donor lungs for a minimum of 180 min. Standard criteria donor lungs underwent EVLP due to logistical indications, whereas marginal donor lungs underwent assessment and reconditioning due to medical indications.

### Inclusion criteria

EVLP was performed with both DBD and DCD donor lungs with either a logistical or medical indication.

Medical indication criteria:

1. Donor pO_2_ < 40 kPa at FiO2 1.0 and PEEP 5 despite recruitment maneuvers.
2. Pulmonary edema, hemorrhage, contusion, or emboli.
3. Extended DCD (eDCD) donor lungs, as defined by an agonal phase > 2 hours.^15^
4. And/or microbiological, bronchoscopic, radiographic, or macroscopic doubtful lung quality.

Logistical indication criteria:

1. Standard criteria donor lungs.
2. Conventional LTx is not possible due to operating room logistics.
3. Cold ischemic time longer than 6 hours (thus extending the total preservation time).

### Exclusion criteria

Unilateral donor lungs were excluded from this study as recipients < 18 years of age.

### Ex vivo lung perfusion

EVLP based on the Toronto protocol was performed as described earlier.^2^, ^4^ The minimum EVLP runtime is 180 min, and the maximum is 360 min. Procedures were terminated either upon meeting LTx acceptance criteria or due to irreversible deterioration of lung function. The assessment was performed every hour by deoxygenating the perfusate using deoxygenation gas (86% CO_2_, 8% CO_2_, and 6% O_2_) and increasing the FiO_2_ to 1.0 for 10 min. Perfusate samples were taken every 30 min and were stored at −80°C until measurements were performed.

### End points

The primary endpoints were the biomarkers for fibrinolysis, coagulation, inflammation, glycocalyx shedding, and endothelial activation. Secondary endpoints were PGD grades 72 hours post-LTx and 1-year survival. PGD was defined and graded as stated in the consensus by the ISHLT.^16^

### Biomarkers

The biomarkers were measured in perfusate samples taken after 90 and 180 min of EVLP. Enzyme-linked immunosorbent Assays (ELISA) were used to measure the following markers according to the manufacturer’s protocol (R&D Systems Europe, Abingdon, UK): uPAR (DY807), PAI-1 (DTSE100) Interleukin-1β (DY201), Interleukin-6 (DY206), Interleukin-8 (DY208), TNF-α (DY210), Hyaluronan (DY3614), Syndecan-1 (DY2780), and VCAM-1 (DY809). Prothrombin F1+2 (Enzygnost F1+2 ELISA Siemens Nederland N.V.) Before actual measurements, all ELISA kits were validated for usage with Steen solution. D-dimer levels were determined using a StaCompact Max 3 (Stago, Leiden, The Netherlands). Measured concentrations were adjusted for the Steen solution refreshment and volume changes during EVLP.

### Statistical analysis

IBM Statistics version 28 (SPSS Inc., Chicago, Ill., USA) was used for statistical analysis and GraphPad Prism 8.0 (GraphPad Software, San Diego, CA) for graphical visualization. Data were checked for normal distribution by Q-Q plots and the Shapiro-Wilk test. Paired and unpaired *t*-tests were used for normal distribution, while Mann-Whitney *U* and Wilcoxon Rank tests were used for non-normally distributed data. For correlation analysis, Point Biserial analysis was performed for binomial data and Spearman for continuous and ordinal data. The log-rank test was used to compare survival distributions. *P*-values < 0.05 are considered statistically significant.

## Results

### Study groups

All EVLP procedures performed between February 2019 and January 2023 were included in this study. This comprises 33 procedures, of which 18 had a logistical (standard criteria donor lungs) and 15 a medical indication (marginal donor lungs) for EVLP. After EVLP, 2 donor lungs in the logistical group and 3 in the marginal group were declined for LTx.

### Donor, recipient, and donor lung characteristics

Significant differences in donor characteristics between the logistical and marginal groups were seen for donor age, female sex, total lung capacity (TLC) and last donor pO_2_. In the marginal group, (persistent) atelectasis appears to be a major influence of low donor pO_2_ (Table 1). For the recipients, the logistical group had a significantly higher lung allocation score (LAS), lower TLC, and longer EVLP duration (Table 2).

**Table 1:** Donor Characteristics of the Logistical and Marginal Groups

Donor	Logistical (*n* = 18)	Marginal (*n* = 15)	*p*-value
Age (years)	62.0 ± 15.3	50.6 ± 12.0	***0.023***
DBD/DCD	13/5	8/7	0.281
Female % (*n*)	77.8% (14)	36.3% (5)	**0.020**
Height (cm)	173.0 ± 8.0	177.9 ± 10.4	0.129
Weight (kg)	77.7 ± 18.8	85.3 ± 15.0	0.206
Predicted TLC (L)	5.9 ± 1.0	6.8 ± 1.2	**0.023**
BMI (kg/m^2^)	25.7 ± 4.4	28.3 ± 3.8	0.178
Mechanical ventilation (days)	4.1 ± 3.6	5.75 ± 5.3	*0.323*
Smoking history % (*n*) Pack years (years)	33.3% (6) 12.2 ± 11.6	56.3% (9) 10.3 ± 7.7	0.266 0.718
DCD WIT (min)	21.0 ± 11.2	21.1 ± 12.6	0.999
Cause of death			
Cerebrovascular			
− Bleeding	15	7	
− Stroke	-	3	
Trauma	2	3	
Anoxia	1	-	
Cardiac arrest	-	2	
Last pO_2_ (kPa)	57.5 ± 13.3	33.89 ± 15.56	***<.001***
Medical indication/possible cause of low pO_2_			
− Unexplained	-	3	
− Atelectasis	-	5	
− Atelectasis with edema	-	3	
− Edema	-	2	
− eDCD + PE	-	2

**Table 2:** Recipient and EVLP Characteristics of the Logistical and Marginal Group

Recipient	Logistical (*n* = 16)	Marginal (*n* = 12)	*p*-value
Age (years)	56.9 ± 7.2	52.25 ± 13.91	0.256
Female % (*n*)	68.8 (11)	25.0 (3)	0.097
Height (cm)	174.8 ± 10.0	177.0 ± 5.5	0.454
Weight (kg)	75.2 ± 16.5	73.9 ± 15.5	0.828
BMI (kg/m^2^)	24.6 ± 5.0	23.6 ± 4.6	0.574
LAS	37.1 ± 5.1	32.7 ± 1.6	***0.002***
FEV_1_ (L/s)	1.3 ± 0.90	1.6 ± 1.1	0.531
TLC (L)	5.5 ± 2.1	7.8 ± 2.7	***0.017***
HLM %	-	17 (2)	0.479
ECMO perioperative % (*n*)	69 (11)	42 (5)	0.452
VA-ECMO % (*n*)	100 (11)	100 (5)	
ECMO postoperative % (*n*) VA-ECMO % (*n*) VV-ECMO % (*n*)	31 (5)80 (4)20 (1)	8 (1)100 (1)-	0.324
CIT I (min)	256.4 ± 49.8	252.2 ± 41.7	0.812
EVLP duration	295.0 ± 45.8	247.6 ± 41.2	***0.009***
CIT II 1st lung (min)	276.3 ± 73.7	243.6 ± 64.8	0.246
CIT II 2nd lung (min)	408.0 ± 100.1	373 ± 80.4	0.356
Lung disease			
Emphysema	7	7	
Pulmonary arterial hypertension	1	-	
Secondary pulmonary arterial hypertension	4	3	
Idiopathic lung fibrosis	4	1	
Cystic fibrosis	-	1	
Conversion rat%	89	80	0.682

DBD, donation after brain death; DCD, donation after circulatory death; eDCD, extended donation after circulatory death (converted from DCD when agonal phase >2 hours)^15^; WIT, warm Ischemia Time; TLC, total lung capacity; BMI, body mass index; PO_2_, arterial partial oxygen pressure at PEEP 5 cmH_2_O and FiO_2_ 1.0; PE, pulmonary emboli. Values are expressed as the mean with standard deviation.

### Fibrinolysis and coagulation

**Figure 1:**
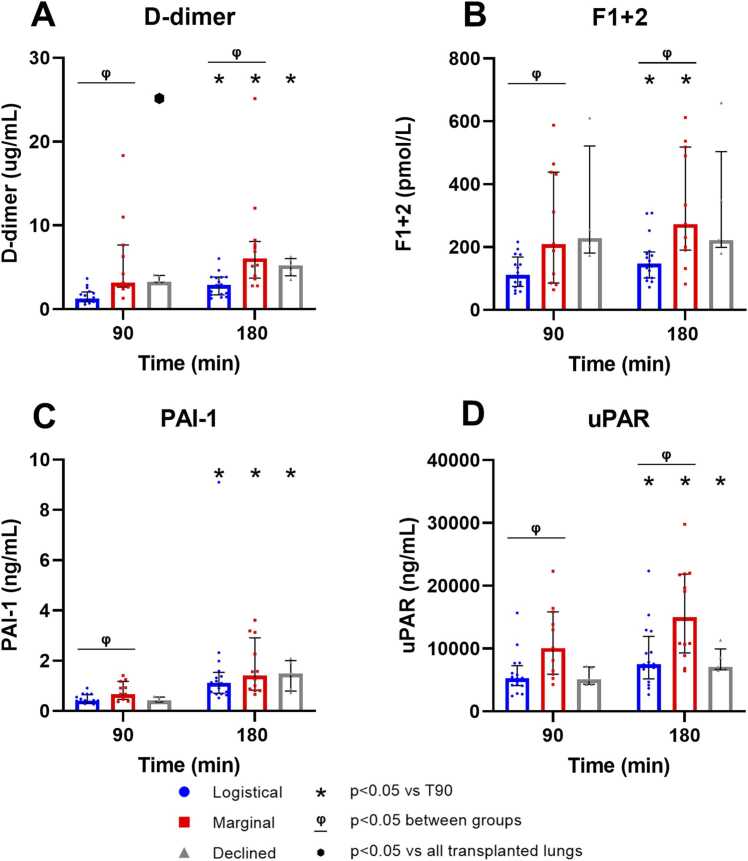
Fibrin degradation and coagulation markers at 90 and 180 min of EVLP. (A) D-dimer (logistical *n* = 16, marginal *n* = 12, declined *n* = 5), (B) Prothrombin Fragment 1+2, (F1+2; logistical *n* = 16, marginal *n* = 11, declined *n* = 5) (C) Plasminogen activator inhibitor-1 (PAI-1; logistical *n* = 16, marginal *n* = 12, declined *n* = 5) and (D) soluble urokinase Plasminogen Activator Receptor (uPAR; logistical *n* = 16, marginal *n* = 12, declined *n* = 5). Individual datapoints are displayed, and values are expressed as median with IQR.

D-dimer, F1+2, PAI-1, and uPAR increased significantly in both logistical and marginal groups, while the amounts of uPAR, d-dimer, and F1+2 at both timepoints were significantly higher in the marginal group (Figure 1, Table S1). Declined donor lungs displayed similar trends, except for F1+2 (Figure 1B; Table S2).

### Pro-inflammatory cytokines

**Figure 2:**
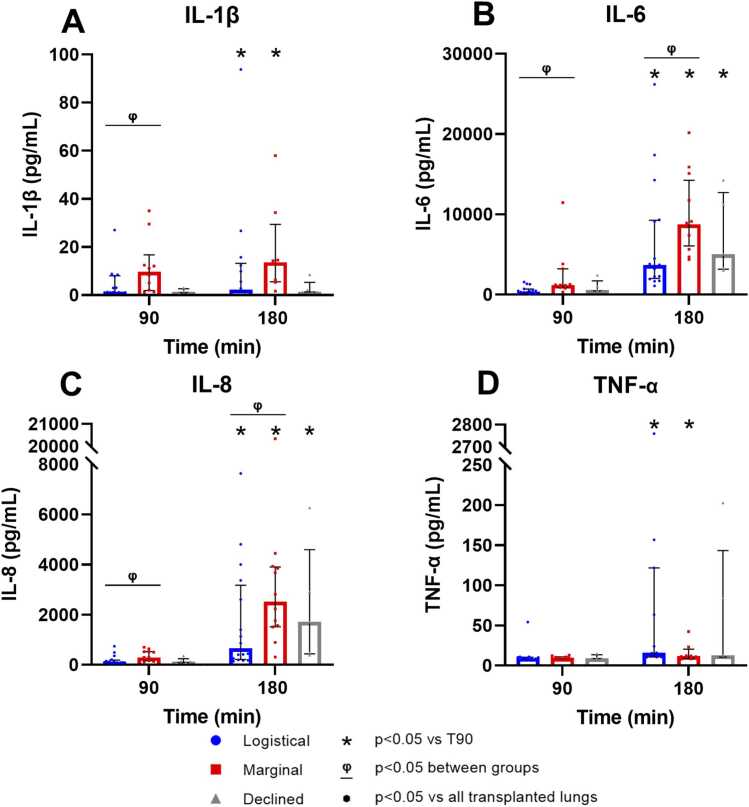
Pro-inflammatory cytokines at 90 and 180 min of EVLP. (A) Interleukin-1β (IL-1β logistical *n* = 15, marginal *n* = 10, declined *n* = 5), (B) Interleukin-6 (IL-6; logistical *n* = 16, marginal *n* = 12, declined *n* = 5), (C) Interleukin-8 (IL-8; logistical *n* = 16, marginal *n* = 12, declined *n* = 5), and (D) Tumor Necrosis Factor-α (TNF- α logistical *n* = 14, marginal *n* = 8, declined *n* = 5). Individual datapoints are displayed, and values are expressed as median with IQR.

The concentration of IL-1β, IL-6, IL-8, and TNF-α increased significantly in the logistical and marginal groups, while the marginal group exhibited significantly higher IL-6 and IL-8 than the logistical group (Figure 2, Table S2). No significant differences were seen between the transplanted and declined donor lungs (Table S2).

### Endothelium

**Figure 3:**
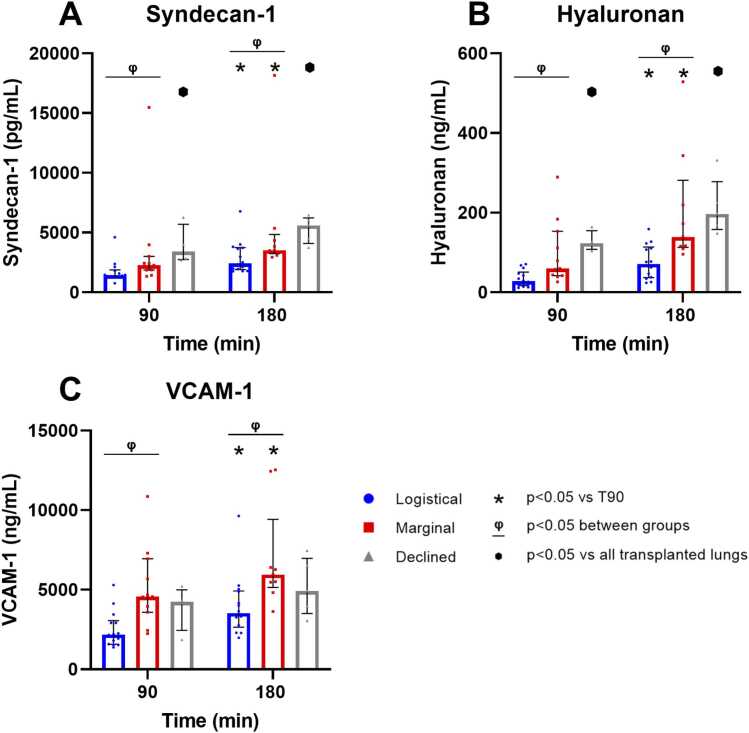
Endothelial glycocalyx degradation products and endothelial cell activation marker at 90 and 180 min of EVLP. (A) Syndecan-1 (logistical *n* = 15, marginal *n* = 11, declined *n* = 5), (B) Hyaluronan (logistical *n* = 15, marginal *n* = 11, declined *n* = 5), and (C) Vascular Cell Adhesion Molecule-1 (VCAM-1; logistical *n* = 15, marginal *n* = 11, declined *n* = 5). Individual datapoints are displayed, and values are expressed as median with IQR.

The marginal group showed significantly higher amounts of endothelial glycocalyx components and the endothelial activation marker VCAM-1 (Figure 3, Table S1). Notably, syndecan-1 and hyaluronan levels were significantly higher in the declined donor lungs when compared to all transplanted donor lungs (Figures 3A and B, Table S2).

### Oxygenation capacity, pulmonary vascular resistance, and weight differences

**Figure 4:**
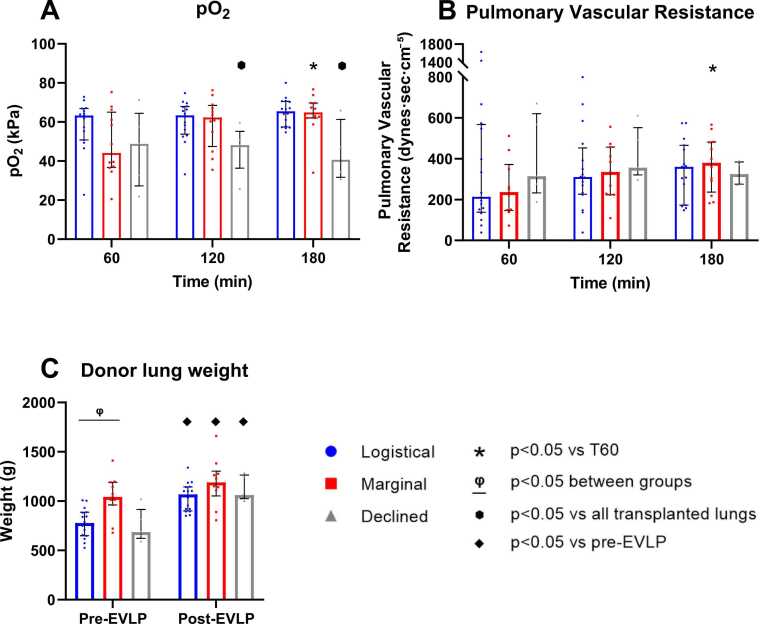
Functional parameters during at 60, 120, and 180 min of EVLP with logistical (*n* = 16), marginal (*n* = 12), and declined (*n* = 5) donor lungs. (A) PO_2_, (B) Pulmonary Vascular Resistance (PVR) and (C) Donor lung weight difference in percentage at post-EVLP compared to pre-EVLP. Individual datapoints are displayed, and values are expressed as median with IQR.

The pO_2_ increased significantly over time in the marginal group, while the pO_2_ at 120 and 180 min in the declined group were significantly lower compared to all transplanted donor lungs (Figure 4A). PVR increased significantly in the marginal group after 180 min but remained comparable to the logistical group (Figure 4B). Both groups gained significant weight. The marginal group had a significantly higher weight pre-EVLP (Figure 4C). Weight post-EVLP did not differ between the logistical and marginal groups (Table 2). No significant differences were observed between the declined and transplanted donor lungs.

### Declined donor lungs

In the logistical and medical groups, 2 and 3 donor lungs were declined for LTx, respectively. Both logistical donor lungs were declined due to insufficient pO_2_, with the combination of extensive pulmonary or alveolar edema. Pathology reported an early stage of diffuse alveolar damage and aspiration-induced granulomatous inflammation, respectively. All declined donor lungs in the marginal group were declined due to insufficient pO_2_ and/or evident pulmonary edema. Pathology reported: 1) nonspecific chronic bronchial inflammation, 2) thrombus in a larger artery of the left lower lobe, and 3) hemorrhagic infarctions with recent thrombi in both right and left lower lobes.

### Correlation EVLP parameters

Higher PVR seems to significantly correlate with increased ΔSyndecan-1 and decreased ΔIL-1β. Additionally, pO_2_ was negatively associated with IL-6 level. Increased levels of (Δ)IL-8, (Δ)TNF-α, and ΔPAI-1 were significantly associated with mortality within the first year (Table S6).

### Clinical outcomes

**Figure 5:**
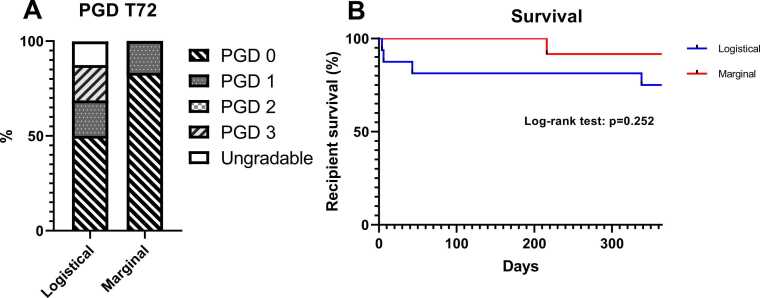
Clinical outcomes. (A) Primary graft dysfunction after 72 hours post-LTx, graded as 0-3 or ungradable according to the ISHLT 2016 PGD consensus statement.^17^ Incidence is expressed as a percentage. (B) Recipient 1 year survival. Survival is expressed as a percentage.

PGD grades 72 h post-LTx did not differ significantly between the logistical and marginal groups (grade 1: 18.7% vs 16.7%, p = 0.763; grade 3: 18.7% vs 0%, p = 0.095). In two recipients, PGD was ungradable due to VA-ECMO (Figure 5A). One-year survival was similar between both groups (Figure 5B). None of the recipients died due to PGD or Chronic Lung Allograft Dysfunction (CLAD). Causes of death in the logistical group were: 1) post-operative cerebral infarction, 2) distributive shock with multiorgan failure, 3) fatal hemoptysis due to a broncho-pulmonary artery fistula, 4) post-transplant lymphoproliferative disorder, and 5) infection with a mucormycosis with extensive (pulmonary) angio-invasion. In the marginal group, one recipient suffered from fatal respiratory insufficiency due to pulmonary Kaposi sarcoma.

## Discussion

In this study, we performed combined measurements of established biomarkers of fibrinolysis, inflammation, glycocalyx shedding, and endothelial activation during EVLP in both standard-criteria and marginal donor lungs. Although most biomarkers increased over time, marginal donor lungs and particularly donor lungs declined for transplantation, demonstrated distinct biomarker profile indicating injury.

We reported earlier that (micro)thrombi were present in the retrograde flush of all donor lungs and even in the second retrograde flush when EVLP was performed.^17^ This suggests that residual thrombi are left inside the donor despite the current flushing protocol does not completely remove (micro)thrombi, leaving some. Both logistical and marginal groups showed significant increases in d-dimer, with higher levels in the marginal group. This indicates endogenous fibrinolysis and also possibly the release of preexisting d-dimer. These findings provide additional rationale for applying standard thrombolytic therapy during EVLP, particularly in marginal donor lungs, for example, with urokinase.^18^ However, we did not observe significant correlations between d-dimer levels and EVLP parameters or clinical outcomes. Interestingly, two of the three declined marginal donor lungs showed thrombi on pathological analysis, suggesting that thrombolytic therapy during EVLP could have been beneficial for these two donor lungs. The F1+2 levels, a marker of coagulation activation, increased significantly during EVLP, with higher levels in the marginal group. This increase likely reflects the release of preexisting F1+2, as thrombi formation is unlikely in heparinized acellular Steen solution. The rise in d-dimer, without an assumed increase in newly formed F1+2, indicates that increased fibrinolysis results from the breakdown of preexisting thrombi.^19^ Likewise, this suggests that marginal lung donors were in a more hypercoagulable state.

To further investigate dysregulation of coagulation and fibrinolysis, we measured uPAR and PAI-1. Fibrinolysis can be stimulated by membrane bound uPAR, which facilitates juxtaposition between urokinase and membrane associated plasminogen.^20^ Soluble uPAR (suPAR) has been associated with increased fibrinolytic activity.^21^ Conversely, PAI-1 can inhibit plasminogen activators, thereby promoting fibrin deposition to seal damaged barriers. Endothelial cells can express uPAR and may attempt to counteract (micro)thrombi by promoting fibrinolysis, as the marginal group showed significantly higher uPAR and d-dimer levels than the logistical group.^22^ Moreover, uPAR and PAI-1 can amplify inflammation through Toll-Like Receptor activated neutrophils, while uPAR also exerts immunomodulatory effects on neutrophil infiltration and recruitment.^20^, ^23^, ^24^ Nevertheless, the exact roles of uPAR and PAI-1 in EVLP remain incompletely elucidated.

Pro-inflammatory cytokines play a crucial role during ischemia-reperfusion injury, which can lead to PGD.^25^ IL-6 and IL-8 are established biomarkers for predicting PGD during EVLP.^26^, ^27^ In our study, the marginal group showed significantly higher concentrations of IL-8 and IL-6, suggesting greater inflammation. Based on the IL-6/IL-8 inflammation score to predict suitability for LTx and PGD during EVLP, the marginal donor lungs would be classified in a higher category than the standard criteria donor lungs. ^27^ However, this was not the highest of the four categories associated with the worst clinical outcomes, which may explain similar PGD grades and 1-year survival. A proportion of marginal donor lungs in the highest inflammation category may have already been declined for EVLP at our center, likely due to selective donor lung acceptance.

Other pro-inflammatory markers, IL-1β and TNF-α, are significantly associated with declined donor lungs after EVLP and mortality.^28^ We did not observe significant differences in IL-1β and TNF-α at 180 min. However, IL-8 and TNF-α levels significantly correlated with 1-year mortality (Table S6). Recently, Boffini et al. showed that using a Cytosorb filter during EVLP reduced IL-6 levels, which were associated with lower in-hospital mortality and higher one-year survival despite a non-significant increase in PGD.^29^ Thus, attenuating inflammatory cytokines with a cytokine absorber during EVLP may be beneficial. In contrast, Noda et al. suggested that circulating leukocytes, rather than pro-inflammatory cytokines, significantly affect lung function during EVLP.^30^ Improving the use of (multiple) leukocyte filters during EVLP could be another approach to reduce inflammation and enhance lung function.

The pulmonary endothelium, with the glycocalyx as the luminal layer, is critical for lung function. Deterioration of the glycocalyx results in increased vascular pressure, endothelial permeability, and increased edema development.^31^ Specifically, the glycocalyx component hyaluronan correlates with decreased dynamic compliance and increased edema.^32^ High levels of endothelial activation markers during EVLP, such as VCAM-1, are linked with PGD.^33^ The marginal group exhibited significantly higher levels of syndecan-1, hyaluronan, and VCAM-1, reflecting greater injury. Declined donor lungs had significantly higher hyaluronan and syndecan-1 levels than all transplanted donor lungs at both time points, consistent with prior pilot studies.^32^, ^34^ This indicates that the endothelial glycocalyx could serve as an early prognostic biomarker for the progression of edema and lung function during EVLP, independent of inflammatory injury mechanisms, as pro-inflammatory cytokine levels did not differ significantly. High levels of syndecan-1 in lung donors and their recipients were significantly associated with PGD development.^35^ Moreover, syndecan-1 has been shown to be a biomarker to predict early allograft dysfunction after transplantation during machine perfusion of donor livers.^36^ The glycocalyx shedding in the declined group likely contributed to lower pO_2_ and a higher nonsignificant increase in weight. Targeting the endothelial glycocalyx through heparinase inhibition, transient heat stress or optimized heparin use may preserve and improve graft function.^37^, ^38^

Two pairs of standard criteria donor lungs were declined after EVLP, although they would normally have been transplanted without EVLP. Donor data, chest radiography, and bronchoscopy showed no abnormalities explaining the deterioration during EVLP. The post-transplant performance of these lungs therefore remains unknown. EVLP may have identified unrecognized graft problems, but this requires confirmation in larger cohorts.^39^

Interestingly, PGD grades and survival were similar between the logistical and marginal groups despite differences in biomarker profiles. We observed similar outcomes in our previous report.^4^ This could suggest that the injury reflected by the marginal group might be reversible rather than indicative of irreversible damage. The recipients of the logistical group, with a significantly higher LAS, showed a non-significantly higher incidence of PGD grade 3 after 72 hours and 1-year mortality despite having a more favorable biomarker profile. However, PGD development is influenced by several factors. Characteristics of the donor, such as PO_2_ and donor lung weight, are associated with a higher incidence of PGD.^9^, ^40^ The logistical group gained significantly more weight during EVLP, but this may be affected by the higher pre-EVLP weight of the marginal group due to a higher presence of pre-existing edema. Also, the recipient LAS may influence clinical outcomes, highlighting the importance of the recipient condition in PGD development.^41^, ^42^ Interestingly, the development of early PGD grade 3 after EVLP tends to resolve more quickly and yield better outcomes than conventional LTx.^43^ A more transient hydrostatic phenotype of PGD could follow after EVLP rather than a later immunological/inflammatory phenotype.^41^ Further research, including biomarker measurements in the recipient, would be required to elucidate lung injury after EVLP and the effect potential therapeutic interventions.

An interesting characteristic in the marginal lung donors is the relatively high prevalence of (persistent) atelectasis (Table 1), which likely contributed to a significantly lower donor pO_2_. Atelectasis resistant to recruitment maneuvers is associated with reduced oxygenation, decreased compliance, increased PVR and shunting.^44^, ^45^ Additionally, airway stress and compression of the (micro)vasculature may induce epithelial and endothelial damage, promoting inflammation.^45^ Impaired flow may cause blood stasis in the (micro)vasculature, facilitating (micro)thrombi formation. Conversely, sudden alveolar recruitment and rapid reperfusion may exacerbate lung injury.^46^ Therefore, (persistent) atelectasis may affect marginal donor lungs and warrants further investigation.

Although this study shows significant results, it has some limitations. It is a retrospective, single-center study with relatively small groups, which may underpower our analysis. The exact impact on clinical outcomes requires further validation in a larger cohort. Due to the relatively short minimum EVLP time of 180 min, the progression of some biomarkers could not have been adequately reflected in the perfusate. This retrospective study was limited to perfusate samples only. Paired tissue analysis, cell-free DNA or Neutrophil Extracellular Traps associated markers could have provided additional validation in terms of the endothelial glycocalyx injury and early innate immune activation.^47^, ^48^ Moreover, paired proteomics or metabolomics analysis could have provided more in-depth molecular injury pathways.^49^, ^50^

## Conclusion

In conclusion, marginal donor lungs appear to have sustained more injury than standard criteria donor lungs as reflected in higher levels of d-dimer, pro-inflammatory cytokines, endothelial activation, and glycocalyx shedding during EVLP. The endothelial glycocalyx shedding seems to be an early prognostic biomarker in declined donor lungs. Interventions targeting thrombolysis, reducing inflammation, and preserving the endothelium and glycocalyx could enhance the quality of donor lungs. This may increase EVLP conversion rates and the lung donor pool. EVLP remains necessary to distinguish between acceptable marginal and too-marginal donor lungs for LTx.

## Disclosure statement

HL is the Chief Scientific Officer for 34Lives, a public benefit organization. ME holds a patent with XVIVO perfusion. All other authors declare no conflict of interest.

## Acknowledgments

Dominique Koerts, Lotte van der Scheer, and Petra Ottens provided excellent skills in ordering, handling, and measuring all perfusate samples. Willie Steenhuis invaluably managed and collected the clinical data with great care.

## Financial support

All authors have no financial disclosures to declare.

## CRediT authorship contribution statement

Michiel Hu (MH), Zhang Zhang (ZZ), Roland Hoffmann (RF), Erik Verschuuren (EV), Tji Gan (TG), Caroline Van De Wauwer (CW), Henri Leuvenink (HL), and Michiel Erasmus (ME). All authors made significant contributions to this work. Methodology: MH, ZZ, ME. Data curation: MH. Formal analysis and interpretation: MH, ZZ, RF, EV, TG, CW, HL, ME. Writing of original draft: MH. All authors revised and provided critical feedback, resulting in the final manuscript.

## Footnotes

Appendix A Supplementary data associated with this article can be found in the online version at doi:10.1016/j.jhlto.2026.100555.
