# Supplementary material for: Biomarkers of standard criteria and marginal donor lungs during ex vivo lung perfusion: A comparative study
Source: JHLT Open. 2026 Apr 4;13:100555. doi: 10.1016/j.jhlto.2026.100555 (PMC13133946; doi:10.1016/j.jhlto.2026.100555)
Supplement: Supplementary file 1 — Supplementary material [file mmc1.docx]

**Supplementary materials**

Biomarkers of Standard Criteria and Marginal Donor Lungs During Ex Vivo Lung Perfusion: A Comparative Study

M.A. Hu MD^1^ Orcid# 0000-0002-7355-099X

Z.L. Zhang MD^1^ Orcid# 0000-0003-2137-7555

R.F. Hoffmann PhD^1^ Orcid# 0000-0003-2473-4124

C.T. Gan MD PhD^2^ Orcid# 0000-0002-1310-4189

E.A.M. Verschuuren MD PhD^2^ Orcid# 0000-0002-6807-6744

C. Van De Wauwer MD PhD^1^ Orcid# [0000-0002-0886-4917](http://orcid.org/0000-0002-0886-4917)

H.G.D. Leuvenink PhD^3^ Orcid# 0000-0001-5036-2999

M.E. Erasmus MD PhD^1^ Orcid# 0000-0002- 6234-2976

*^1^Department of Cardiothoracic Surgery, University Medical Center Groningen, Groningen, Netherlands*

*^2^ Department of Pulmonary Diseases and Lung Transplantation, University Medical Center Groningen, Groningen, Netherlands*

*^3^ Department of Surgery, University Medical Center Groningen, Groningen, Netherlands*

Table of contents

Supplementary tables

Table S12

Table S23

Table S34

Table S45

Table S56

Table S1. Comparison of the measured biomarkers of the transplanted donor lungs in the logistical (n=16) and medical (n=12) group during EVLP at 90, 180 min of EVLP and the delta value (90-180 min). Values are expressed as median with IQR.

|  | **Logistical** | | **Medical** | |  |
| --- | --- | --- | --- | --- | --- |
|  | **Median** | **IQR** | **Median** | **IQR** | **P-value** |
| **D-dimer T90** | 1.3 | 0.9 - 2.1 | 3.2 | 2.7 - 7.7 | ***<.001*** |
| **D-dimer T180** | 2.9 | 1.7 - 3.8 | 6.1 | 3.7 - 8.1 | ***0.002*** |
| **Δ D-dimer** | 1.1 | 0.7 - 1.7 | 1.5 | 1 - 2.7 | 0.323 |
| **F1+2 T90** | 111.8 | 74.9 - 168.3 | 209.7 | 85.8 - 438.4 | ***0.038*** |
| **F1+2 T180** | 147.4 | 101.7 - 184.3 | 272.7 | 190.1 - 518.7 | ***0.010*** |
| **Δ F1+2** | 28.6 | 20 - 44.6 | 63.0 | 21.9 - 86.8 | 0.167 |
| **PAI-1 T90** | 0.4 | 0.3 - 0.6 | 0.7 | 0.5 - 1.2 | ***0.017*** |
| **PAI-1 T180** | 1.1 | 0.7 - 1.5 | 1.4 | 0.8 - 2.9 | 0.265 |
| **Δ PAI-1** | 0.7 | 0.4 - 0.9 | 0.9 | 0.3 - 2 | 0.675 |
| **uPAR T90** | 5281.2 | 4105.9 - 7272.5 | 10067.9 | 5906.3 - 15861.2 | ***0.009*** |
| **uPAR T180** | 7504.6 | 5196.6 - 11951.1 | 14969.4 | 9318.2 - 21845 | ***0.020*** |
| **Δ uPAR** | 2446.0 | 1278 - 4887.8 | 4345.0 | 2704 - 8023 | ***0.048*** |
| **IL-1β T90** | 1.3 | 1.1 - 8 | 9.7 | 1.9 - 16.7 | ***0.026*** |
| **IL-1β T180** | 2.2 | 1.3 - 13.2 | 13.6 | 5.5 - 29.3 | 0.065 |
| **Δ IL-1β** | 1.1 | 0.2 - 6.9 | 2.8 | 0 - 4.6 | 0.945 |
| **IL-6 T90** | 349.7 | 227.4 - 684.3 | 1189.4 | 882.8 - 3217.8 | ***0.004*** |
| **IL-6 T180** | 3656.6 | 2012.1 - 9254.5 | 8743.1 | 6065.4 - 14247.3 | ***0.026*** |
| **Δ IL-6** | 3439.0 | 1806.5 - 8324 | 7650.0 | 5305 - 11424 | 0.054 |
| **IL-8 T90** | 98.6 | 88.4 - 191.9 | 293.3 | 164.3 - 526.3 | ***0.008*** |
| **IL-8T180** | 650.6 | 209.1 - 3174.8 | 2522.1 | 1520.9 - 3906.1 | ***0.046*** |
| **Δ IL-8** | 558.9 | 116.6 - 2751.2 | 2375.4 | 1141.8 - 3518.8 | 0.063 |
| **TNF-α T90** | 9.2 | 8.2 - 10.5 | 9.5 | 8.7 - 11 | 0.597 |
| **TNF-α T180** | 15.8 | 10.7 - 121.6 | 11.6 | 8.9 - 20.5 | 0.148 |
| **Δ TNF-α** | 6.7 | 2.3 - 114.3 | 1.4 | 0.8 - 8.5 | ***0.031*** |
| **Syndecan-1 T90** | 1420.2 | 1320.2 - 1871.1 | 2258.3 | 1860.7 - 3000.1 | ***0.012*** |
| **Syndecan-1 T180** | 2426.4 | 1900.3 - 3745.6 | 3527.7 | 3240.2 - 4849.8 | ***0.038*** |
| **Δ Syndecan-1** | 883.1 | 481.9 - 1520.4 | 1438.1 | 1286.9 - 1710.9 | 0.057 |
| **Hyaluronan T90** | 27.8 | 16.8 - 50.7 | 59.9 | 41.8 - 153.2 | ***0.007*** |
| **Hyaluronan T180** | 71.1 | 36.9 - 113.8 | 138.8 | 112.5 - 281.4 | ***0.003*** |
| **Δ Hyaluronan** | 31.9 | 18.1 - 48.4 | 93.3 | 57.8 - 151.3 | ***<.001*** |
| **VCAM-1 T90** | 2178.3 | 1565.6 - 3051.9 | 4556.1 | 3578.9 - 6940.6 | ***0.001*** |
| **VCAM-1 T180** | 3537.0 | 2648 - 4915.2 | 5940.3 | 5139.8 - 9417.8 | ***0.003*** |
| **Δ VCAM-1** | 1082.1 | 821.7 - 1659 | 1547.5 | 893.9 - 1859.3 | 0.367 |

IQR, Interquartile Range; uPAR, urokinase Plasminogen Activator Receptor; PAI-1, Plasminogen Activator Inhibitor-1; F1+2, Prothrombin Fragment 1+2; IL-6, Interleukin-6; IL-8, Interleukin-8; TNF-α, Tumor Necrosis Factor-α; IL-1, Interleukin-1; VCAM-1, Vascular Cellular Adhesion Molecule-1; T90, 90 min of EVLP; T180, 180 min of EVLP

Table S2. Comparison of the measured biomarkers of all transplanted (n=28) and declined (n=5) donor lungs during EVLP at 90, 180 min of EVLP and the delta value (90-180 min). Values are expressed as median with IQR.

|  | **Transplanted** | | **Declined** | |  |
| --- | --- | --- | --- | --- | --- |
|  | **Median** | **IQR** | **Median** | **IQR** | **p-value** |
| **D-dimer T90** | 2.1 | 1.2 - 3.2 | 3.3 | 3.2 - 4.0 | ***0.040*** |
| **D-dimer T180** | 3.8 | 2.3 - 5.8 | 5.2 | 4 - 6.0 | 0.248 |
| **Δ D-dimer** | 1.2 | 0.7 - 1.9 | 2.0 | 0.3 - 2.4 | 0.979 |
| **F1+2 T90** | 135.3 | 80.5 - 209.7 | 228.4 | 181.1 - 521.9 | 0.068 |
| **F1+2 T180** | 178.5 | 125.1 - 306.9 | 222.1 | 199.1 - 503.7 | 0.113 |
| **Δ F1+2** | 36.0 | 20.9 - 75.3 | 48.1 | 23.3 - 82.7 | 0.637 |
| **PAI-1 T90** | 0.5 | 0.4 - 0.7 | 0.4 | 0.4 - 0.6 | 0.377 |
| **PAI-1 T180** | 1.2 | 0.8 - 1.9 | 1.5 | 0.8 - 2.0 | 0.802 |
| **Δ PAI-1** | 0.7 | 0.3 - 1.1 | 1.1 | 0.4 - 1.5 | 0.436 |
| **uPAR T90** | 5906.3 | 4308.0 - 10610.6 | 5095.2 | 4302.8 - 7098.6 | 0.452 |
| **uPAR T180** | 9336.5 | 6882.6 - 18170.0 | 7083.3 | 6692.0 - 9956.0 | 0.315 |
| **Δ uPAR** | 2962.0 | 1730.0 - 5219.0 | 2590.0 | 1374.5 - 3571.0 | 0.312 |
| **IL-1β T90** | 3.0 | 1.1 - 10.5 | 1.3 | 1.1 - 2.6 | 0.250 |
| **IL-1β T180** | 6.0 | 1.5 - 14.9 | 1.3 | 1.3 - 5.3 | 0.092 |
| **Δ IL-1β** | 2.2 | 0.2 - 5.3 | 0.2 | 0.1 - 4.1 | 0.226 |
| **IL-6 T90** | 718.8 | 285.8 - 1251.6 | 572.5 | 465.3 - 1723.1 | 0.697 |
| **IL-6 T180** | 6505.3 | 3293.0 - 11125.0 | 5044.7 | 3173.3 - 12724.4 | 0.960 |
| **Δ IL-6** | 5305.0 | 2730.0 - 8686.0 | 4472.0 | 2708 - 11001.5 | 0.938 |
| **IL-8 T90** | 149.7 | 91.7 - 496.1 | 120.2 | 90.3 - 246.5 | 0.467 |
| **IL-8T180** | 1536.2 | 407.0 - 3803.5 | 1711.4 | 439.7 - 4592.3 | 0.802 |
| **Δ IL-8** | 1335.4 | 312.4 - 3336.2 | 1574.0 | 335.1 - 4368.6 | 0.802 |
| **TNF-α T90** | 9.3 | 8.7 - 10.6 | 9.0 | 8.8 - 13.4 | 0.732 |
| **TNF-α T180** | 12.2 | 10.7 - 42.5 | 12.6 | 10.6 - 143.4 | 0.749 |
| **Δ TNF-α** | 3.4 | 1.6 - 30.3 | 2.9 | 1.6 - 142.0 | 0.839 |
| **Syndecan-1 T90** | 1788.7 | 1352.2 - 2390.6 | 3410.5 | 2739.5 - 5694.3 | ***0.014*** |
| **Syndecan-1 T180** | 3390.4 | 2125.3 - 3843.7 | 5598.9 | 4092.3 - 6226.3 | ***0.013*** |
| **Δ Syndecan-1** | 1286.9 | 710.5 - 1620.8 | 1611.2 | 542.4 - 2874.0 | 0.434 |
| **Hyaluronan T90** | 42.1 | 25.4 - 69.1 | 123.3 | 107.9 - 154.8 | ***0.016*** |
| **Hyaluronan T180** | 109.4 | 62.9 - 138.8 | 196.8 | 157.9 - 277.7 | ***0.013*** |
| **Δ Hyaluronan** | 51.0 | 28.9 - 89.3 | 64.9 | 35.8 - 188.6 | 0.477 |
| **VCAM-1 T90** | 2923.2 | 2039.1 - 4612.3 | 4244.0 | 2437.8 - 4989.4 | 0.569 |
| **VCAM-1 T180** | 4816.3 | 3324.9 - 5940.3 | 4922.9 | 3500.1 - 6974.1 | 0.610 |
| **Δ VCAM-1** | 1297.0 | 860.5 - 1819.1 | 1690.9 | 860.6 - 2223.5 | 0.394 |

IQR, Interquartile Range; uPAR, urokinase Plasminogen Activator Receptor; PAI-1, Plasminogen Activator Inhibitor-1; F1+2, Prothrombin Fragment 1+2; IL-6, Interleukin-6; IL-8, Interleukin-8; TNF-α, Tumor Necrosis Factor-α; IL-1, Interleukin-1; VCAM-1, Vascular Cellular Adhesion Molecule-1; T90, 90 min of EVLP; T180, 180 min of EVLP

|  |  | **D-dimer** | **Δ D-dimer** | **F1+2** | **Δ F1+2** | **PAI-1** | **Δ PAI-1** | **uPAR** | **Δ uPAR** |
| --- | --- | --- | --- | --- | --- | --- | --- | --- | --- |
| **Age (years)** | ρ | -.222 | -.148 | -.200 | -.243 | 0.079 | 0.178 | ***-.500*** | ***-.419*** |
|  | p-value | 0.215 | 0.417 | 0.273 | 0.187 | 0.662 | 0.329 | ***0.003*** | ***0.017*** |
| **Height (cm)** | ρ | 0.033 | -.142 | 0.022 | -.121 | 0.271 | 0.175 | 0.265 | 0.185 |
|  | p-value | 0.855 | 0.437 | 0.904 | 0.518 | 0.128 | 0.338 | 0.135 | 0.312 |
| **Weight (kg)** | ρ | ***0.394*** | 0.224 | ***0.484*** | 0.227 | ***0.534*** | ***0.460*** | ***0.463*** | ***0.355*** |
|  | p-value | ***0.023*** | 0.218 | ***0.005*** | 0.219 | ***0.001*** | ***0.008*** | ***0.007*** | ***0.046*** |
| **BMI** | ρ | ***0.480*** | ***0.354*** | ***0.361*** | 0.156 | ***0.355*** | 0.344 | 0.281 | 0.277 |
|  | p-value | ***0.005*** | ***0.047*** | ***0.043*** | 0.401 | ***0.043*** | 0.054 | 0.113 | 0.126 |
| **Last PO_2_ (kPa)** | ρ | ***-.509*** | -.236 | -.294 | -.108 | -.162 | -.066 | ***-.558*** | ***-.512*** |
|  | p-value | ***0.002*** | 0.194 | 0.102 | 0.564 | 0.368 | 0.721 | ***<.001*** | ***0.003*** |
| **Pack years** | ρ | 0.165 | -.203 | 0.215 | -.270 | 0.159 | -0.008 | 0.320 | 0.154 |
|  | p-value | 0.572 | 0.506 | 0.481 | 0.372 | 0.588 | 0.979 | 0.265 | 0.615 |
| **Donor TLC (L)** | ρ | 0.061 | -.146 | 0.034 | -.128 | 0.295 | 0.174 | 0.326 | 0.221 |
|  | p-value | 0.736 | 0.426 | 0.852 | 0.492 | 0.096 | 0.340 | 0.064 | 0.223 |
| **Ventilation (days)** | ρ | 0.369 | 0.145 | 0.203 | -.116 | -.156 | -.168 | 0.252 | 0.182 |
|  | p-value | 0.053 | 0.471 | 0.310 | 0.563 | 0.429 | 0.403 | 0.197 | 0.362 |
| **DCD WIT (min)** | ρ | -.042 | 0.006 | 0.018 | 0.117 | ***0.782*** | ***0.855*** | ***-.673*** | ***-.636*** |
|  | p-value | 0.907 | 0.987 | 0.960 | 0.765 | ***0.008*** | ***0.002*** | ***0.033*** | ***0.048*** |
| **Female sex** | rpb | -.021 | 0.111 | -.080 | 0.090 | -.044 | 0.020 | ***-.508*** | ***-.395*** |
|  | p-value | 0.906 | 0.547 | 0.664 | 0.632 | 0.810 | 0.915 | ***0.003*** | ***0.025*** |
| **DCD** | rpb | ***0.480*** | ***0.376*** | ***0.411*** | 0.101 | 0.330 | 0.299 | ***0.355*** | ***0.486*** |
|  | p-value | ***0.005*** | ***0.034*** | ***0.019*** | 0.587 | 0.061 | 0.097 | ***0.043*** | ***0.005*** |

Table S3. Correlation analysis between the donor characteristics and uPAR, PAI-1, d-dimer and F1+2 levels of all donor lungs measured at 180 min and the delta value between 90 and 180 min of EVLP.

uPAR, urokinase Plasminogen Activator Receptor; PAI-1, Plasminogen Activator Inhibitor-1; F1+2, Prothrombin Fragment 1+2; Δ, delta value between 90 and 180 min; BMI, Body Mass Index; TLC, Total Lung Capacity; DCD, Donation after Circulatory Death; DBD, Donation after Brain Death; WIT, donor Warm Ischemia Time; ρ, Spearman’s correlation coefficient; rpb; point biserial correlation coefficient

Table S4. Correlation analysis between the donor characteristics and IL-1β, IL-6, IL-8 and TNF-α levels of all donor lungs measured at 180 min and the delta value between 90 and 180 min of EVLP.

IL-6, Interleukin-6; IL-8, Interleukin-8; TNF-α, Tumor Necrosis Factor-α; IL-1, Interleukin-1; Δ, delta value between 90 and 180 min; BMI, Body Mass Index; TLC, Total Lung Capacity; DCD, Donation after Circulatory Death; DBD, Donation after Brain Death; WIT, donor Warm Ischemia Time; ρ, Spearman’s correlation coefficient; rpb; point biserial correlation coefficient

|  |  | **IL-1β** | **Δ IL-1β** | **IL-6** | **Δ IL-6** | **IL-8** | **Δ IL-8** | **TNF-α** | **Δ TNF-α** |
| --- | --- | --- | --- | --- | --- | --- | --- | --- | --- |
| **Age (years)** | ρ | -.290 | -.113 | -.254 | -.222 | ***-.355*** | ***-.319*** | -.119 | 0.011 |
|  | p-value | 0.142 | 0.582 | 0.153 | 0.222 | ***0.043*** | ***0.071*** | 0.580 | 0.960 |
| **Height (cm)** | ρ | 0.324 | 0.028 | 0.268 | 0.240 | 0.142 | 0.100 | -.116 | -.074 |
|  | p-value | 0.099 | 0.893 | 0.132 | 0.186 | 0.430 | 0.580 | 0.588 | 0.736 |
| **Weight (kg)** | ρ | 0.166 | -.093 | 0.277 | 0.294 | ***0.389*** | ***0.367*** | -.034 | -.106 |
|  | p-value | 0.407 | 0.652 | 0.118 | 0.102 | ***0.025*** | ***0.036*** | 0.873 | 0.630 |
| **BMI** | ρ | -.010 | -.142 | 0.172 | 0.150 | 0.275 | 0.271 | 0.085 | -.068 |
|  | p-value | 0.961 | 0.489 | 0.339 | 0.412 | 0.122 | 0.128 | 0.692 | 0.758 |
| **Last PO_2_ (kPa)** | ρ | ***-.382*** | -.145 | ***-.504*** | ***-.481*** | ***-.517*** | ***-.493*** | -.102 | 0.114 |
|  | p-value | ***0.049*** | 0.480 | ***0.003*** | ***0.005*** | ***0.002*** | ***0.004*** | 0.636 | 0.605 |
| **Pack years** | ρ | 0.413 | -.298 | ***0.562*** | ***0.603*** | 0.353 | 0.342 | 0.243 | 0.109 |
|  | p-value | 0.235 | 0.403 | ***0.036*** | ***0.029*** | 0.216 | 0.232 | 0.498 | 0.763 |
| **Donor TLC (L)** | ρ | ***0.396*** | 0.063 | 0.319 | 0.278 | 0.199 | 0.155 | -.089 | -.052 |
|  | p-value | ***0.041*** | 0.761 | 0.070 | 0.124 | 0.267 | 0.390 | 0.679 | 0.814 |
| **Ventilation (days)** | ρ | 0.075 | -.094 | 0.208 | 0.232 | 0.207 | 0.210 | 0.126 | 0.008 |
|  | p-value | 0.740 | 0.677 | 0.287 | 0.245 | 0.290 | 0.284 | 0.607 | 0.973 |
| **DCD WIT (min)** | ρ | -.467 | 0.167 | -.115 | -.006 | -.006 | 0.030 | -.262 | -.357 |
|  | p-value | 0.174 | 0.668 | 0.751 | 0.987 | 0.987 | 0.934 | 0.531 | 0.432 |
| **Female sex** | rpb | ***-.528*** | -.368 | -.337 | -.261 | -.315 | -.153 | 0.182 | 0.191 |
|  | p-value | ***0.005*** | 0.064 | 0.055 | 0.150 | 0.074 | 0.396 | 0.394 | 0.384 |
| **DCD** | rpb | 0.065 | -.025 | ***.390*** | ***.358*** | ***0.438*** | ***0.479*** | 0.297 | 0.316 |
|  | p-value | 0.749 | 0.903 | ***0.025*** | ***0.044*** | ***0.011*** | ***0.005*** | 0.159 | 0.142 |

Table S5. Correlation analysis between the donor characteristics and syndecan-1, hyaluronan, VCAM-1 levels of all donor lungs measured at 180 min and the delta value between 90 and 180 min of EVLP.

uPAR, urokinase Plasminogen Activator Receptor; PAI-1, Plasminogen Activator Inhibitor-1; F1+2, Prothrombin Fragment 1+2; Δ, delta value between 90 and 180 min; BMI, Body Mass Index; TLC, Total Lung Capacity; DCD, Donation after Circulatory Death; DBD, Donation after Brain Death; WIT, donor Warm Ischemia Time; ρ, Spearman’s correlation coefficient; rpb; point biserial correlation coefficient

|  |  | **Syndecan-1** | **Δ Syndecan-1** | **Hyaluronan** | **Δ Hyaluronan** | **VCAM-1** | **Δ VCAM-1** |
| --- | --- | --- | --- | --- | --- | --- | --- |
| **Age (years)** | ρ | -.148 | 0.099 | ***-.434*** | ***-.427*** | -.362 | -.278 |
|  | p-value | 0.451 | 0.629 | ***0.021*** | ***0.029*** | 0.058 | 0.169 |
| **Height (cm)** | ρ | 0.075 | -.058 | -.026 | 0.070 | 0.246 | 0.055 |
|  | p-value | 0.705 | 0.779 | 0.896 | 0.734 | 0.206 | 0.791 |
| **Weight (kg)** | ρ | 0.340 | 0.085 | 0.218 | 0.140 | 0.362 | 0.106 |
|  | p-value | 0.076 | 0.678 | 0.264 | 0.496 | 0.058 | 0.605 |
| **BMI** | ρ | ***0.453*** | 0.256 | 0.358 | 0.187 | 0.312 | 0.112 |
|  | p-value | ***0.016*** | 0.207 | 0.061 | 0.360 | 0.106 | 0.587 |
| **Last PO_2_ (kPa)** | ρ | -.309 | ***-.453*** | ***-.570*** | ***-.597*** | ***-.629*** | ***-.482*** |
|  | p-value | 0.110 | ***0.020*** | ***0.002*** | ***0.001*** | ***<.001*** | ***0.013*** |
| **Pack years** | ρ | 0.365 | 0.444 | 0.274 | 0.146 | 0.492 | 0.170 |
|  | p-value | 0.300 | 0.199 | 0.444 | 0.688 | 0.148 | 0.638 |
| **Donor TLC (L)** | ρ | 0.084 | -.063 | 0.036 | 0.150 | 0.292 | 0.128 |
|  | p-value | 0.669 | 0.760 | 0.857 | 0.465 | 0.132 | 0.535 |
| **Ventilation (days)** | ρ | 0.111 | 0.369 | 0.199 | 0.206 | 0.402 | 0.164 |
|  | p-value | 0.614 | 0.091 | 0.364 | 0.358 | 0.057 | 0.467 |
| **DCD WIT (min)** | ρ | -.200 | -.500 | ***-.794*** | ***-.786*** | -.467 | -.690 |
|  | p-value | 0.580 | 0.207 | ***0.006*** | ***0.021*** | 0.174 | 0.058 |
| **Female sex** | rpb | -.150 | 0.055 | -.258 | -.300 | ***-.420*** | -.350 |
|  | p-value | 0.446 | 0.790 | 0.185 | 0.136 | ***0.026*** | 0.080 |
| **DCD** | rpb | 0.244 | 0.004 | 0.217 | 0.057 | 0.364 | 0.181 |
|  | p-value | 0.211 | 0.984 | 0.267 | 0.781 | 0.057 | 0.377 |
